# Optofluidic Tweezers: Efficient and Versatile Micro/Nano-Manipulation Tools

**DOI:** 10.3390/mi14071326

**Published:** 2023-06-28

**Authors:** Yuchen Zhu, Minmin You, Yuzhi Shi, Haiyang Huang, Zeyong Wei, Tao He, Sha Xiong, Zhanshan Wang, Xinbin Cheng

**Affiliations:** 1 Institute of Precision Optical Engineering, School of Physics Science and Engineering, Tongji University, Shanghai 200092, China; 2211195@tongji.edu.cn (Y.Z.); 1953799@tongji.edu.cn (H.H.); weizeyong@tongji.edu.cn (Z.W.); wangzs@tongji.edu.cn (Z.W.); chengxb@tongji.edu.cn (X.C.); 2 MOE Key Laboratory of Advanced Micro-Structured Materials, Shanghai 200092, China; 3 Shanghai Institute of Intelligent Science and Technology, Tongji University, Shanghai 200092, China; 4 Shanghai Frontiers Science Center of Digital Optics, Shanghai 200092, China; 5 National Key Laboratory of Advanced Micro and Nano Manufacture Technology, Shanghai Jiao Tong University, Shanghai 200240, China; minminyou@sjtu.edu.cn; 6 School of Automation, Central South University, Changsha 410083, China

**Keywords:** optical tweezers, optofluidics, multifunctional manipulation, bioparticles

## Abstract

Optical tweezers (OTs) can transfer light momentum to particles, achieving the precise manipulation of particles through optical forces. Due to the properties of non-contact and precise control, OTs have provided a gateway for exploring the mysteries behind nonlinear optics, soft-condensed-matter physics, molecular biology, and analytical chemistry. In recent years, OTs have been combined with microfluidic chips to overcome their limitations in, for instance, speed and efficiency, creating a technology known as “optofluidic tweezers.” This paper describes static OTs briefly first. Next, we overview recent developments in optofluidic tweezers, summarizing advancements in capture, manipulation, sorting, and measurement based on different technologies. The focus is on various kinds of optofluidic tweezers, such as holographic optical tweezers, photonic-crystal optical tweezers, and waveguide optical tweezers. Moreover, there is a continuing trend of combining optofluidic tweezers with other techniques to achieve greater functionality, such as antigen–antibody interactions and Raman tweezers. We conclude by summarizing the main challenges and future directions in this research field.

## 1. Introduction

In 1986, Authur Ashkin reported his groundbreaking work on the trapping of a single microparticle stably in three dimensions using a focused laser beam. Thereafter, this technical implementation became known as “optical tweezers” (OTs) [1,2]. The essence of the manipulation of particles by OTs is to change the motion of the particles through the transfer of momentum [2]. Since their initial implementation, OTs have played an increasingly important role in exploring nonlinear optics [3], soft-condensed-matter physics [4,5], molecular biology [6,7], and analytical chemistry [8]. Owing to their great contributions to the cooling and trapping of atoms with laser light, Steven Chu, Claude Cohen-Tannoudji and William D. Phillips were awarded the Nobel Prize in Physics in 1997 [9,10,11]. It was only in 2018 that Authur Ashkin earned the Nobel Prize in Physics for his pioneering work in and applying OTs to biological systems [2,12].

Over the last 30 years, OTs have evolved beyond classical single-beam traps, and they are now widely used in capturing [6,13,14], sorting [15], transporting [16,17,18,19], and enriching [20,21,22] particles, including biological particles. However, despite their significant advances, OTs in stationary environments still face some challenging limitations that should be taken into account. Firstly, the speed of the manipulated particles is limited. Secondly, the beam needs to move in order to reach the target particles. Thirdly, the functionality of the system is simplistic, as it is only capable of capturing, moving, and localizing particles, and there is a lack of efficient screening of different particles and other functions. Finally, the volume of the manipulated solution is also limited, typically in the order of picoliter [23].

**Figure 1:**
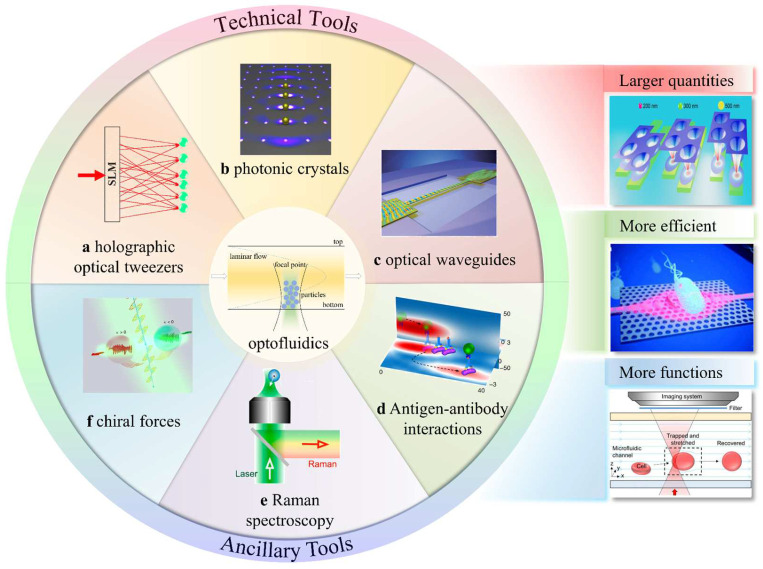
Brief schematic diagram of optofluidic tweezers, some modern optofluidic-tweezer technologies, and the advantages of optofluidic tweezers: (**a**) HOTs, reproduced with permission from [39], copyright @ 2020 Optica Publishing Group; (**b**) photonic crystals, reproduced with permission from [40], copyright @ 2015 American Chemical Society; (**c**) optical waveguides, reproduced with permission from [41], copyright @ 2008 Springer Nature; (**d**) antigen/antibody interactions, reproduced with permission from [35], copyright @ 2018 Springer Nature; (**e**) Raman spectroscopy, reproduced with permission from [42], copyright @ 2019 American Chemical Society; (**f**) chiral forces, reproduced with permission from [43], copyright @ 2020 Springer Nature; larger quantities, reproduced with permission from [44], copyright @ 2020 American Chemical Society; more efficient, reproduced with permission from [45], copyright @ 2013 The Royal Society of Chemistry; more functions, reproduced with permission from [33], copyright @ 2020 The Royal Society of Chemistry.

Microfluidics refers to the utilization of 2D and 3D microscaled channels for the transportation, separation, sorting, and cultivation of various particles in a single tiny chip [2]. Optofluidics, on the other hand, pertains to the fusion of photonics and microfluidics. So far, review articles have been published on optofluidics in microstructured optical fibers [24], the interaction between light and flowing liquids [25], optofluidic bioimaging [26], thermo-optofluidic applications [27], and microdroplet-based optofluidic enzyme monitoring [28], which are integrated with microfluidics. Optical forces, particularly from the spatial light focusing created by various optical components, are commonly employed in microfluidic applications to capture and manipulate suspended particles within microchannels [2]. Microfluidic chips offer numerous advantages in the field of micro/nano manipulation, such as the high throughput of fluids and particles, the ease of generating diverse environments, the ability of particles to move between different environments, the precise control of flow fields, and automatic control via software [23,25,29,30]. When combined with OTs, microfluidics can increase operational efficiency and enable more functions, including the efficient sorting of particles [31], the rapid formation of colloids [32], the analysis of the mechanical responses of cells [33], and the examination of adsorption mechanics [34], as illustrated in Figure 1. It is particularly important to note that the realization of more of the functionalities of optofluidic tweezers may depend on their joint use with other technologies. A considerable amount of work in this area has emerged in recent years, such as the combination with antigen-antibody interactions [35] and Raman optical tweezers [36,37,38]. This demonstrates the excellent compatibility of optofluidic tweezers with many other techniques. This is a key part of recent advances in the field of optofluidic tweezers and is described in detail.

This paper starts with static OTs, and then outlines the most recent developments and opportunities associated with optofluidic tweezers. This paper emphasizes various research achievements in the use of OTs in the past five years based on different technologies, such as holography, photonic crystals, and optical waveguides. Next, auxiliary experimental methods combined with OTs to realize more functions, such as Raman spectroscopy, chiral optical forces, and antigen–antibody interactions, are presented. Furthermore, we explore how to improve the performance of optofluidic tweezers and identify their new prospects combined with these technologies. Based on current research, we highlight the shortcomings that are still present in current optofluidic systems. Lastly, the future direction and boundless possibilities of optofluidic tweezers are discussed.

## 2. Static Optical Tweezers

Before summarizing the development of static OTs, it is necessary to briefly mention the method used to calculate the forces of OTs on an object. Based on the conservation of momentum, when the intensity and direction of light change, part of the light momentum may be transferred to suspended particles, leading to particle motion. The accurate mathematical description of these momentums and forces requires a rigorous electromagnetic theory, which involves simulating the interaction between incident electromagnetic waves and microscopic particles. In most cases, this process requires complex modeling and a significant amount of calculation. However, some simpler theoretical methods provide accurate results, with the caveat that the characteristic sizes of particles are much smaller or much larger than the light wavelength [39]. Depending on the relative size of the particle, there are three methods for calculating the optical force. When the particle radius *r* is much larger than the optical wavelength λ (r>10λ) and the diffraction effect can be ignored, the geometric optics are sufficiently accurate to calculate the optical force [39,46,47,48,49,50,51,52,53]. When the particle size is sufficiently small to be approximated by electric or magnetic dipoles, a sufficiently accurate set of resolved results can be obtained through a dipole-approximation regime [39,54,55,56,57,58]. When the particle’s dimension is comparable to the wavelength (r ~ λ), advanced numerical simulation methods should be used to solve the vector full-wave electromagnetic scattering, for which the generalized Lorentz–Mie theory is a commonly used electromagnetic model [2]. There are several computational methods available for generalized Lorentz–Mie theory, including the finite element [59,60], the finite-difference time-domain [61,62,63], discrete dipole approximation [64], and the T-matrix [65,66,67,68]. The discussions here focus only on the most common scenarios for optofluidic tweezers. Specifically, only spherical particles are considered in the dipole-approximation model, and only homogeneous media are considered in the electromagnetic model. For more detailed derivations and more complex cases, please refer to other specific papers [2,35,46,47,48,49,50,51,52,53,54,55,56,57,58,59,60,61,62,63,64,65,66,67,68].

### 2.1. Geometric Model

The geometric model provides sufficient accuracy in describing light–matter interactions when the object is much larger than the wavelength. In the geometric model, a light beam can be viewed as comprising an indivisible set of rays with a specific direction, intensity, and polarization [39]. Each ray in this model carries a power $P_{i}=P_{in}/N$, where $P_{in}$ is the total incident power of the beam and N is the number of individual rays. The rays propagate in straight lines, neglecting diffraction effects, reflecting and refracting at the interface according to Fresnel’s formula [47,48,49,50].

**Figure 2:**
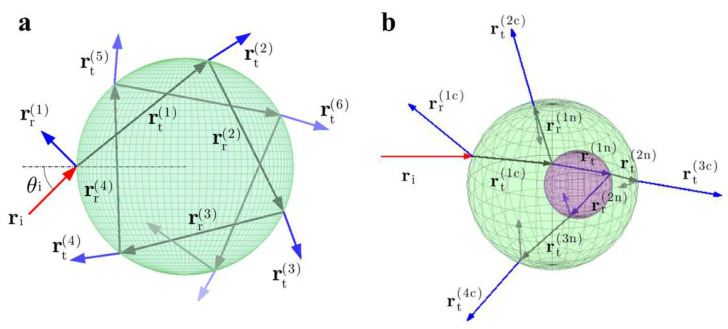
Graphical representation (**a**) and grid approximation (**b**) of optical force, where $r_{i}$, $r_{t}^{\left(n\right)}$, and $r_{r}^{\left(n\right)}$ refer to the incident ray, the emitted ray, and the reflected ray, respectively. Reproduced with permission from [51]. Copyright @ 2015 Optical Society of America.

When a ray $r_{i}$ with power $P_{i}$, enters a particle, multiple iterations of reflection and refraction occur before all of the rays are emitted from the particle. As a consequence, the total optical force acting on the particle by the ray (represented by $F_{\mathrm{ray}}$) should be calculated according to the Fresnel formulas, as shown in Figure 2 [51]:

(1) $$F_{\mathrm{ray}}=\frac{n_{i}P_{i}}{c}r_{i}-\frac{n_{i}P_{r}}{c}r_{r}^{\left(1\right)}-\sum_{n=2}^{+\infty }\frac{n_{t}P_{t,n}}{c}r_{t}^{\left(n\right)},$$

where the unit vectors $r_{i}$, $r_{r}^{\left(n\right)}$, and $r_{t}^{\left(n\right)}$ represent the direction of the incident ray, the nth reflected ray, and the nth refracted ray, respectively, and $r_{r}^{\left(1\right)}$ refers to the first reflected ray. The total force exerted on the particle by all the rays is $F_{\mathrm{ray}}^{m}$. If there are multiple rays interacting with a particle, the total force can be calculated by summing the forces generated by each ray [50]:

(2) $$F_{\mathrm{sum}}=\sum_{m}F_{\mathrm{ray}}^{m}.$$

The geometric model allows the definition of incident rays, which can then be used in conjunction with the ray-tracing method to determine the position and direction of reflected and transmitted rays [52,53]. Building upon classical geometric theory, Callegari et al. developed the OT software package, which is designed for computing optical forces and moments [51]. This toolbox not only provides an excellent computing environment for theoretical calculations, but also enables researchers without programming experience to perform efficient theoretical analyses.

### 2.2. Dipole-Approximation Model

For small particles, dipole approximation can be used to analytically calculate optical forces with acceptable levels of error [54]. The following derivation requires the use of Maxwell’s stress tensor, but the final form of the equations is very simple. Consider a particle with permittivity $\varepsilon_{p}$, magnetic permeability $\mu_{p}$, refractive index $n_{p}={\left(\varepsilon_{p}\;\mu_{p}\right)}^{1/2}$, and radius r embedded in a homogeneous, non-dispersive medium. The refractive index of the background medium is represented by $n={\left(\varepsilon \;\mu \right)}^{1/2}$, and its electromagnetic parameters are  ε and μ. The electric and magnetic polarization rates of the spheres can be expressed through Mie coefficients, denoted by $a_{v}$ and $b_{v}$, respectively [55]:

(3) $$a_{v}=\frac{\mu m^{2}j_{v}\left(mx\right){\left[xj_{v}\left(x\right)\right]}^{\prime }-\mu_{p}j_{v}\left(x\right){\left[mxj_{v}\left(mx\right)\right]}^{\prime }}{\mu m^{2}j_{v}\left(mx\right){\left[xh_{v}^{\left(1\right)}\left(x\right)\right]}^{\prime }-\mu_{p}h_{v}^{\left(1\right)}\left(x\right){\left[mxj_{v}\left(mx\right)\right]}^{\prime }},$$

(4) $$b_{v}=\frac{\mu_{p}j_{v}\left(mx\right){\left[xj_{v}\left(x\right)\right]}^{\prime }-\mu j_{v}\left(x\right){\left[mxj_{v}\left(mx\right)\right]}^{\prime }}{\mu_{p}j_{v}\left(mx\right){\left[xh_{v}^{\left(1\right)}\left(x\right)\right]}^{\prime }-\mu h_{v}^{\left(1\right)}\left(x\right){\left[mxj_{v}\left(mx\right)\right]}^{\prime }},\;$$

where $x=nk_{0}r$ is the normalized particle radius and $k_{0}=\frac{\omega }{c}$ is the wave vector in vacuum with angular frequency ω and light velocity c. The relative refractive index of the particle is $m=n_{p}\;/n$, where $n_{p}$ and n are the refractive indices of the particle and background medium, respectively. The spherical Bessel function $j_{v}$ and Hankel function $h_{v}^{\left(1\right)}$ of order v (an integer from 1 to infinity) are used in the derivations, which are performed with respect to the argument (e.g., $j_{v}\left(nx\right)\prime \;=d\;\left[j_{v}\left(nx\right)\right]/d\;\left(nx\right)$). The total scattered field can be obtained by summing all the harmonic components from ν=1 to ν=+∞.

When the first electromagnetic Mie coefficients, $a_{1}$ and $b_{1}$, are much larger than the others (i.e., $\left|a_{1}\right|\gg \left|a_{v1}\right|$ and $\left|b_{1}\right|\gg \left|b_{v1}\right|$) and v1≫2, the polarization rate of the particle is dominated by the first electric and magnetic components. In this case, the other components can be neglected, leading to what is known as the dipole-approximation condition [39]. 

**Figure 3:**
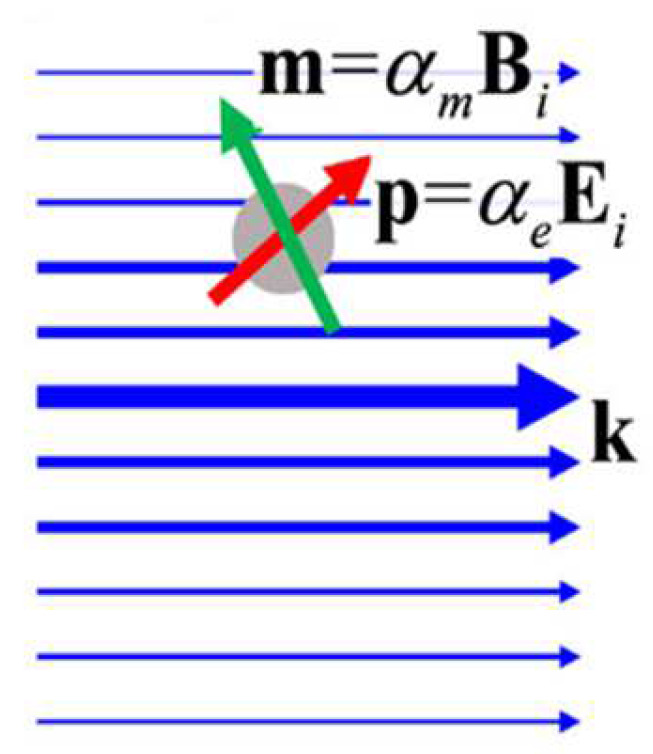
Dipole-approximation model, where the responses of the object to the incident light are represented by an electric dipole p and a magnetic dipole m, reproduced with permission from [39]. Copyright @ 2020 Optica Publishing Group.

Under this condition, the scattered fields correspond to the radiation fields of the induced electric dipole moment p and magnetic moment m, as shown in Figure 3. 

Using the integration of Maxwell’s stress tensor, the time average of the optical force can be derived in the same manner as in previous papers [56,57,58]:

(5) ⟨F⟩=12Rep∇⊗Ei*+m∇⊗Bi*−2k43μεp×m*,

where ⊗ represents the binary product, * is the complex conjugate symbol, and k is the wavenumber, with k=nω/c, ω, c representing the frequency and the speed of light, respectively. The first and second terms, respectively, represent the forces caused by the induced electric and magnetic dipoles, while the third term reflects the forces arising from the interaction between the electric and magnetic dipoles. When the electric and magnetic moments are proportional to the incident field (i.e., $p=\alpha_{e}E_{i}$ and $m=\alpha_{m}B_{i}$, where $\alpha_{e}=3i\varepsilon a_{1}∕\left(2k^{3}\right)$ and $\alpha_{m}=3ib_{1}∕\left(2\mu k^{3}\right)$), and kr ≪ 1 (i.e., the Rayleigh limit), the total time-averaged optical force can be expressed in terms of the polarizabilities. While the equation above would ideally be clearer, these simplifying conditions make it possible to express the force in a more straightforward way [56]:

(6) $$\langle F\rangle =\langle F_{e}\rangle +\langle F_{m}\rangle +\langle F_{e-m}\rangle .$$

The three terms at the right of the equation can be calculated by the following equations:

(7) ⟨Fe⟩=14Reαe∇E2+k2nImαeReE×B*+12ImαeImE*⋅∇E,

(8) ⟨Fm⟩=14Reαm∇B2+kn2ImαmReE×B*+12ImαmImB*⋅∇B,

(9) ⟨Fe−m⟩=−k43μεReαeαm*ReE×B*−Imαeαm*ImE×B*.

### 2.3. Electromagnetic Model

When the particle size is comparable to the wavelength of the light, a rigorous electromagnetic theory can be used to obtain the optical force. The generalized Lorentz–Mie theory is a commonly used electromagnetic model for these calculations [2]. There are several computational methods available for the generalized Lorentz–Mie theory, including the finite element [59,60], the finite-difference time-domain [61,62,63], discrete dipole approximation [64], and the T-matrix [65].

The T-matrix calculation is considered the most mathematically rigorous method and is the focus of this section [66]. In 1965, Waterman introduced the T-matrix as a scattering theory, which was used to solve problems for single or multiple uniform, dispersive, and arbitrarily shaped particles. With the development of solution platforms, T-matrix calculation has become one of the most widely used tools for analyzing particle behavior under optical forces [67,68].

In a homogeneous dielectric, the force density (f) in a unit volume acted upon by electromagnetic phenomena should be expressed by Lorentz’s law [69]:

(10) f=ρE+j×B,

where j, B, E, and ρ represent the current density (A/m), magnetic flux density (T), electric field intensity (N/C) and charge volumetric density ($C/m^{3}$), respectively. It is worth noting that while the forces on particles in a non-homogeneous medium are complex, most studies on and applications of microfluidic tweezers focus on homogeneous or nearly homogeneous fluids. Consequently, the equation for homogeneous media provided above is more commonly used. This same principle applies to the other analogs discussed in this section. According to the law of the conservation of momentum, the momentum flux through the surface of the dielectric can be obtained by integrating the Maxwell stress tensor T. This allows the total force acting on the particle to be expressed as a matrix in Equation (11):

(11) $$F={\int \int }_{s}T\cdot dA-\varepsilon \mu \frac{d}{dt}{\int \int \int }_{V}SdV.$$

The matrix form T can be expressed as

(12) $$\overset{=}{T}=\varepsilon \left[EE-\frac{1}{2}E\cdot E\overset{=}{I}\right]+\frac{1}{\mu }\left[BB-\frac{1}{2}B\cdot B\overset{=}{I}\right],$$

where $\overset{=}{I}$ represents the second-order unit tensor. In the far field, the radial components of the electric and magnetic fields become negligible, allowing the integral of the total force to be expressed in the form of Equation (13):

(13) $$F=\int \int rT_{rr}r^{2}sin\theta d\theta d\phi .$$

Furthermore, the time average of the complex amplitude of the optical force can be expressed using Equation (14):

(14) $$F=-\frac{1}{2}\int \int \hat{r}\left(\varepsilon {\left|E_{0}\right|}^{2}+\mu {\left|H_{0}\right|}^{2}\right)r^{2}sin\theta d\theta d\phi .$$

Over the last few years, numerous software and toolboxes for OTs have been developed based on various theories. Using the flexible programming framework of MATLAB, many researchers have successfully developed it into commercial software, such as the graphical user interface to eliminate positional crosstalk between the output channels created by Hansen’s team [70], the computational platform for HOTs to characterize metal nanoparticles created by Dienerowitz et al. [71], the position-resolved computational tool for particle-tracking signals created by Taylor et al. [72], and the MATLAB toolbox to generate beam patterns with specific phase and amplitude created by Lenton et al. [73]. 

### 2.4. Forms of Static Optical Tweezers

**Figure 4:**
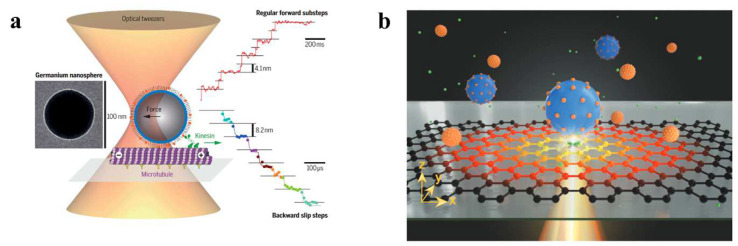
(**a**) Far-field OTs. The colored curves indicate the variation of the center-of-mass position of trapped germanium nanospheres with time, and the black horizontal lines indicate the displacement steps. Reproduced with permission from [75]. Copyright @ 2021 American Association for the Advancement of Science. (**b**) Near-field OTs. The large blue balls indicate the target particles to be captured, while the small orange balls indicate the other particles. Reproduced with permission from [105]. Copyright @ 2022 John Wiley & Sons, Inc.

When operating in static environments, OTs can be categorized into far-field OTs (free-space OTs) and near-field OTs, based on the working regions of the electromagnetic field. Far-field OTs are available in the following forms: single-beam OTs (as shown in Figure 4a), dual-beam OTs, and HOTs. Single-beam OTs were initially used by Ashkin for single-virus capture in the 1980s [6]. Modern single-beam OTs are widely used to capture and move single particles, such as viruses [21], cells [74], protein motors [75], and even animal tissues [76]. However, single-beam OTs often do not meet the increasingly diverse needs of researchers. In biomedical applications, double-beam OTs are widely deployed to stretch DNA or proteins. In this method, each individual OT captures one particle, while each particle attaches to one end of the DNA. The distance between the two beams of light can be increased to stretch the DNA [77,78,79]. On the other hand, HOTs increase the dimensionality of the manipulated particles by a new degree of freedom [80]. By using spatial light modulators, HOTs can create three-dimensional light fields, enabling the manipulation of particles in three dimensions [81,82,83]. Additionally, HOTs enable the construction of some exotic beams, which provide new control functions. For example, the Airy beam can move particles along a curve [84,85,86], the Bessel beam can capture particles in different planes at the same time [87,88], and the vortex beam can make particles follow their orbits [89,90,91,92].

The convergence effect of free-space beams is limited due to the diffraction of light waves, resulting in small gradient forces. When capturing biological particles at the nanoscale, such as viruses, the required laser energy often exceeds 100 mW [21]. Such high-energy lasers may potentially damage biological particles and limit their application in real-life scientific scenarios. Near-field OTs have been developed with the aim of overcoming the limitations of light diffraction, including photonic crystal tweezers [93,94,95], surface-plasmon tweezers [13,96,97,98,99], and fiber-optic-probe tweezers [100,101,102,103]. Photonic crystal tweezers offer good light control and confinement ability due to their precise energy distribution on the surface, allowing them to break the light-diffraction limit. Compared with far-field OTs, photonic crystal tweezers require minimal laser power to capture particles with high efficiency. Surface-plasmon tweezers are also effective in breaking the light-diffraction limit and are widely used to capture fine biological particles, such as nanoparticles and proteins. However, all-dielectric OTs are more favorable for manipulating biological particles to avoid thermal damage from metal-induced light absorption. The use of OTs with fiber probes has gained attention due to their good light-convergence capabilities and their ability to move easily to almost any position. Optoelectronic tweezers (OTETs) exploit the thermosensitive migration of charged ions in a light-induced temperature field to create a local electric field for trapping charged particles (as shown in Figure 4b) [104]. These OTETs require laser power about 100 times lower than that required by conventional OTs, presenting a new low-power platform for manipulating particles of various materials, sizes, and shapes [105]. Furthermore, OTETs have already been successfully used in colloidal assembly [106], printing [107], and cell trapping [108,109].

If the particles captured by the optical tweezers are surrounded by a gas, they are subjected to photophoretic force due to the temperature difference because of the low thermal conductivity of the gas [110]. The photophoretic force is caused by the interaction of gas molecules with the surface of the particle when the surface temperature of the particle is not homogeneous. If the surfaces of the particles are inhomogeneous in terms of accommodable space and/or temperature, each part of the surface absorbs different levels of radiation flux, thus creating a temperature difference. Gas molecules impinge and reflect on the surface of a particle with accommodation, and when the temperature of the particle surface is non-uniform, the gas molecules transfer part of their momentum to the particle, thus generating a force. The photophoretic force on a particle surrounded by a gas is usually more than three orders larger than the optical force on the particle, and is not negligible [111,112,113]. However, in liquids, the photophoretic forces are generally negligible due to the short free range and high thermal conductivity of the liquid. So far, we have not found any applications of photophoretic forces in optofluidic tweezers.

After over 30 years of development, significant progress has been made in OTs. Control methods for OTs have evolved from traditional optical extinction force and gradient force to the intriguing optical pulling forces opposite to the direction of light propagation and optical lateral forces perpendicular to the direction of light propagation. Control accuracy has progressed from the microscale to the nanoscale, and the type of object that can be controlled has extended from traditional dielectric/metal particles to droplets, proteins, bacteria, and other particles ranging from the micron to the nanometric scale.

## 3. Optofluidic Tweezers

Despite great advances made in static OT techniques, they are limited in several ways. Firstly, only a few particles can be manipulated, typically singly or in multiples. Secondly, static OTs capture particles with low efficiency, and often require continuous observations prior to capture. Thirdly, it is difficult to capture specific particles from a solution. Moreover, the amount of solution that can be manipulated is low, usually in the order of picoliters. Finally, the function is simple, usually for capture and movement only [23].

As mentioned previously, microfluidic chips offer several advantages, such as larger fluid volumes, different environments, and finely controlled flow fields, which static OTs lack. Optofluidic tweezers combine the technologies of OTs with microfluidics and are expected to enhance traditional OT technology for more practical applications. 

Beyond this, optofluidic tweezers can be combined with other techniques, such as antigen–antibody interactions and Raman spectroscopy, to enable more features and applications. In particular, Brownian force, which is prevalent and particularly significant for microscopic particles in a medium, is, in most cases, much smaller than the capture force provided by OTs [114]. Compared with the typically faster fluid motion, Brownian motion is often insignificant in optofluidic OTs. There are, however, some examples of the use of Brownian motion. When particles are captured by optical tweezers, these captured particles are subjected to optical forces, photophoretic forces, fluid forces, and Brownian forces. If the particle is spherical and only the translation is taken into consideration, the following equation is available:

(15) $$m\ddot{x}=F_{\mathrm{OTs}}+F_{\mathrm{pho}}+F_{\mathrm{flu}}+F_{B},$$

where *m* represents the mass of the particle, $\ddot{x}$ represents the acceleration of the particle, and $F_{\mathrm{OTs}}$, $F_{\mathrm{pho}}$, $F_{\mathrm{flu}}$, and $F_{B}$ represent the optical forces, photophoretic forces, fluid forces, and Brownian forces, respectively. It should be noted that the above equation is only applicable to spherical particles. Extra terms are induced when the particle is non-spherical and experiences optical torque and complex drag force, leading it to undergo complex kinetics in the light field. Normally, Equation (15) is sufficiently accurate for spherical particles in optical tweezers, while more complete discussions, including rotation can be found in [115,116]. In general, the modeling of optical and photophoretic forces is greatly influenced by the experimental conditions and, therefore, cannot be given a uniform mathematical form. However, in some specific cases, if detailed numerical values of the optical and photophoretic forces can be calibrated, the motions of particles can be derived from the calculation [111]. Conversely, Brownian motion can also be quantified by fitting the equation of motion to the particles after obtaining sufficiently accurate data in the experiment. For example, OTs can be employed to trap colloidal particles, enabling the investigation of the rapid temporal fluctuations in their Brownian motion [114]. The Brownian motion of particles captured by OTs can be utilized to determine the masses of the particles [111]. In addition, Shi et al. used the Brownian motion of particles to achieve the hopping of these particles between different optical traps for screening purposes [35]. This is an innovative attempt to exploit Brownian motion in optofluidic tweezers and demonstrates the potential of optofluidic tweezers in combination with other techniques. The following sections describe recent research advances using optofluidic tweezers according to different technologies.

### 3.1. Holographic Optical Tweezers

A single OT is capable of trapping and manipulating only one particle at a time. However, researchers often require the ability to control multiple particles simultaneously. The conventional approach to this task involves the use of galvanometer scanning or multi-beam coupling to generate multiple optical traps. While this technique does generate a limited number of traps, it falls short in terms of flexibility and complexity. HOTs offer a solution to these limitations. By incorporating a diffractive element, such as a spatial light modulator, a diffraction-beam splitter is placed at the image plane to convert a single input beam into multiple beams. each of which forms a separate optical trap. This beam splitter can take the form of a computer-generated hologram, and the resulting trapping patterns are referred to as HOTs [80,82,117,118]. These elements modulate the incident wavefront, thereby obtaining the desired light field in the objective’s focal region to capture and manipulate particles. In contrast to conventional OTs, HOTs can produce large arrays of arbitrarily arranged and distributed point-like optical traps to capture multiple particles simultaneously. Additionally, computer programming permits independent control over each optical trap, enabling complex dynamic manipulation. Holographic tweezers are also capable of generating optical traps with specialized modes, such as Laguerre–Gauss, Bessel, and Airy beams, by adjusting the incident wavefront. In recent years, significant progress has been made in combining HOTs with microfluidics, with applications such as multi-particle manipulation and bulk particle screening becoming more widespread.

**Figure 5:**
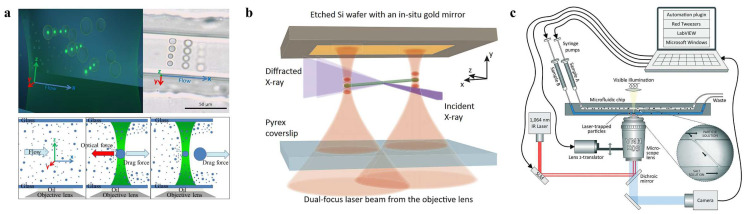
(**a**) HOTs drive colloid generation, reproduced with permission [32]. Copyright @ 2019 Royal Society of Chemistry. (**b**) Driving multiple droplets at the same time, reproduced with permission from [123]. Copyright @ 2020 NAS. (**c**) HOTs control columnar particles, reproduced with permission from [124]. Copyright @ 2021 The Royal Society of Chemistry.

The main advantage of HOTs is the ability to create 3D optical fields, allowing particle manipulation in 3D and the manipulation of multiple particles at the same time. In recent years, researchers have increasingly used these tweezers to manipulate particles at the micro and nano scale. Schmitz et al. created a technique combining dynamic HOTs, fluorescence microscopy, and a specially designed microfluidic system for the construction and detection of bionic actin networks in 2008 [119]. In 2009, Cordero et al. demonstrated new operations on droplets in microchannels using dynamic HOTs [120]. These operations included sorting, re-ordering, and storage, demonstrating the flexibility of HOTs. In 2015, Bolognesi et al. used HOTs to manipulate the construction of a 3D nanofluidic network consisting of several droplets connected by stable oil filaments [121]. The experimental results provided ample evidence of the functional versatility of HOTs through actively controlling the emulsion salinity and temperature, adjusting the interfacial tension of droplets, and providing an environment for in situ characterization. In 2019, Lasnoy et al. proposed a method with which to continuously control polydimethylsiloxane colloid formation based on directional coalescence induced by optical traps within microfluidic channels, as shown in Figure 5a [32]. They demonstrated the controlled formation of PDMS colloids with diameters between 1 and 14 μm by varying the flow rate and laser intensity. This method produced not only a single monodisperse system, but any combination of sizes desired. This technique provides an easy-to-operate method for the quantitative formation of colloids of a specific size. 

In addition, the combination of HOTs with microfluidics can control the particles under the required conditions, providing a stable and suitable environment for the determination of other physical parameters. Gao et al. demonstrated that dynamic HOTs can manipulate individual micron-scale anisotropic particles in a microfluidic environment with the precision and stability required for X-ray Bragg diffraction experiments, thus functioning as optical goniometers (Figure 5b) [122]. Multiple standing-wave focal points can be formed simultaneously and manipulated independently by random mask encoding, so that captured particles can be precisely manipulated in three dimensions. In 2020, Chupeau et al. used a combination of HOTs and microfluidics to study the escape acceleration of Brownian particles with an optimal potential profile [123]. In 2021, Ward et al. proposed an integrated instrument, consisting of microfluidics, HOTs, image processing, and automation software, which could measure a large number of individual pairwise viscous interactions between paired colloidal particles (Figure 5c) [124]. The chemical environment surrounding the particles is exchanged in situ with a “controlled environment” solution, providing a new environment for viscosity measurements within the microfluidic channel. This allows researchers to study a variety of “one-at-a-time” particle interactions within differing chemical environments. The term “one-at-a-time” refers to the measurement of only one change in microscopic particles at a time, under precise control. Unlike more traditional system-wide measurements, which usually give only average results, “one-at-a-time” gives more detailed results directly at the microscopic scale. Ward et al.’s experiments showed that less than 100 μL of emulsion sample is required for each set of 500 paired measurements. This technique provides a new approach to the study of various “one-at-a-time” particle interactions.

### 3.2. Photonic-Crystal Optical Tweezers

Photonic crystals are a novel mechanism for controlling light, based on the concept of photonic band gaps. The electronic band gap is simulated in semiconductors, where the atomic lattice exhibits a periodic potential for electron propagation. Due to the Bragg-like diffraction of atoms, gaps periodically open in the allowed energy range, preventing electrons from propagating in any direction. Large differences in the constituent media’s dielectric constants can generate many of the same phenomena as those observed with the electrons’ atomic potential [125,126]. Therefore, it is crucial to design photonic crystals with a complete photonic band gap, corresponding to a frequency range that prevents the presence of light inside the crystal. This dispersion relationship has an energy-band structure called the photonic energy-band structure. Photonic crystals are composed of materials with low refractive indices periodically located within materials with high refractive indices. These materials can be alternately arranged into specific geometries to produce modulated light and to form a photonic band gap, which is the most fundamental feature of photonic crystals. Photons with energy in the photonic band gap do not enter the crystal, thus enabling wavelength selection. When constructing OTs using photonic crystals, the wavelength selectivity and localization of photonic crystals allow photonic crystal OTs to capture target particles accurately and efficiently.

**Figure 6:**
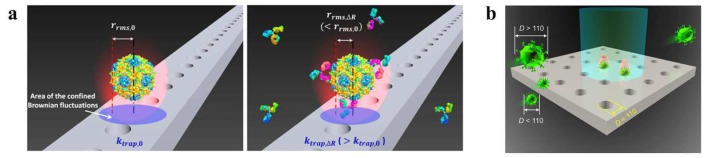
(**a**) One-dimensional photonic-crystal OTs control virus, reproduced with permission from [127]. Copyright @ 2015 Springer Nature. (**b**) Two-dimensional photonic crystal OTs control virus, reproduced with permission from [18]. Copyright @ 2022 Wiley-VCH GmbH.

Because of their precise wavelength selectivity and localization, photonic crystal OTs, can provide greater light intensity with low laser power. Photonic-crystal tweezers have unparalleled advantages in manipulating biological particles, especially living organisms such as viruses, bacteria, etc. In 2013, van Leest et al. used photonic crystals with laser powers of 1 mW or less to create locally enhanced swift fields in an optofluidic chip to capture individual *B. subtilis* and *E. coli* [42]. The experiment overcame the difficulty of the low refractive index of *B. subtilis* and *E. coli* with respect to water and achieved high efficiency, providing a viable option for immobilizing individual biological objects for light-microscopy studies. In 2015, Kang et al. experimentally demonstrated that one-dimensional photonic crystals can provide greater optical power with lower laser energy, leading to the more efficient capture of viruses at the single-particle level [127]. The captured viruses can bind to antibodies, and the number of antibodies bound to the virus can be determined by analyzing the Brownian motion of the virus, as shown in Figure 6a. In 2022, Shi et al. proposed a novel all-dielectric Si_3_N_4_ nanopore OT array with stronger light-field localization and a deeper potential well, as shown in Figure 6b [18]. This technology generates optical forces that overcome the effects caused by dragging forces in fluids of low velocity. Because the material does not absorb light, the photothermal effect is reduced, preserving the activity of biological particles. As a result, versatile control over low-flux unmodified viruses can be achieved by adjusting the size and power of the laser, allowing individual viruses’ capture, transport, and localization, as well as the selective isolation of a large number of viruses of a specific size in the nanopore for separation and purification. In 2023, graphene-nanoribbon-based optofluidic tweezers were proposed to manipulate and classify biological particles with radii of less than 2.5 nm [128]. This design regulates the optical forces of nanoparticles in the vertical direction by varying the gate bias connecting the graphene and the gap distance between the main channel and the graphene nanoribbon. Under the combined effects of fluidic and optical forces, the particles finally enter the corresponding exit branch. This design allows the sorting of particles with radii of less than 2.5 nm and the classification of particles according to their refractive indices. Furthermore, it enables the sorting and precise manipulation of diverse viruses and target particles in liquid environments with an exceptional accuracy, of 1 nm. This achievement suggests a promising avenue for the advancement of nanobiosensors in the future.

The mode field within the photonic-crystal cavity can also be used to excite fluorescence to construct a two-photon or multi-photon microscopy system. Descharmes et al. demonstrated a resonant optical trapping mechanism based on a two-dimensional hollow photonic crystal cavity that exploits the resonant nature of the photonic crystal cavity and the field overlap of the trapped specimen [129]. The experiments achieved the permanent trapping of individual 250 and 500 nm particles with sub-mW power, allowing the detection of particles during their trapping and the retrieval of information about them. This mechanism also demonstrated the ability to individually address multiple cavities on a single photonic-crystal device. Therisod et al. reported the optical trapping of living bacteria in a two-dimensional hollow silicon photonic-crystal cavity [130]. This structure allows the Gram-type differentiation of bacteria at the single-cell scale in a fast, label-free, and non-destructive manner.

### 3.3. Waveguide Optical Tweezers

A waveguide is a channel in an optical device that allows the propagation of light, functioning similarly to a wire in an electronic device. As with optical fibers, waveguides make use of total internal reflection to restrict the light in the channel, thereby confining the energy of light to a certain region. If the region is sufficiently small, the waveguide can exert force to micro- and nanoparticles, restricting their motion. 

**Figure 7:**
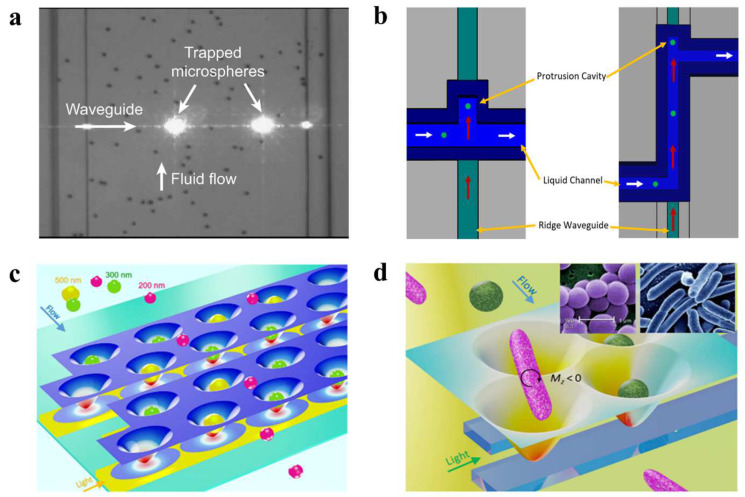
(**a**) Waveguide captures a single polystyrene particle, reproduced with permission from [131]. Copyright @ 2010 Optical Society of America. (**b**) Waveguide-driven sorting of particles, reproduced with permission from [132]. Copyright @ 2022 MDPI. (**c**) Waveguide OT-sorting nanoparticles, reproduced with permission from [44]. Copyright @ 2020 American Chemical Society. (**d**) Waveguide OTs capture bacteria by shape, reproduced with permission from [134]. Copyright @ 2019 American Chemical Society.

When the waveguide is combined with OTs, it can control the direction of particle motion, thus providing an additional dimension of control over particles and improving the efficiency and freedom of OT manipulation. Yang et al. successfully captured polystyrene particles and RNA particles tens of nanometers in diameter using a grooved waveguide of a similar size [17]. Optical scattering forces were then used to drive the particles to move linearly within the groove. However, as the near-field range was limited to only a few hundred nanometers and the thickness of the microchannel containing the grooved waveguide reached 5 microns, most of the particles were not within the effective capture range. The capture rate did not exceed 25% because the capture process relied on the random movement of particles towards the effective range of the near field. Nitkowski et al. demonstrated the simultaneous optical manipulation and analysis of microscale particles in a microfluidic channel, as shown in Figure 7a [131]. All the measurements were performed in-plane, providing an integrated optofluidic platform for lab-on-a-chip biosensing applications. Walker et al. demonstrated an optofluidic device using optical scattering and gradient forces to trap particles in a microchannel with a 300-nm-thick membrane, as shown in Figure 7b [132]. The radiation pressure of the light inside the waveguide pushes the particles into a protruding cavity, which isolates them from the liquid flow. This mechanism serves as a particle sorter and concentrator, increasing the concentration of particles in the small protruding cavity. After particles’ isolation, they can be analyzed by various mechanisms.

Adjacent waveguides, designed using precise field calculations, can be coupled to produce a large number of adjacent subwavelength-scale spots (potential wells) with similar optical-field distributions for each spot. This emerging method creates potential well arrays, overcoming the limitations of traditional techniques, such as holography, which struggle to form uniform subwavelength-scale spot arrays. This method has become a general technique in contemporary OT research. In 2009, Kühn et al. described a fully planar, waveguide-based particle trap that used active feedback to eliminate current-on-chip optical-force-trap drawbacks [133]. The optical force overcomes Brownian motion and residual liquid flow, allowing not only the detection of particles, but also the collection of additional information on internal processes, such as cell-division rates, protein expression, viral infection, binding, replication, and other biological functions without damaging the particles. In 2020, Shi et al. proposed a dual waveguide near-field OT-array configuration, as shown in Figure 7c [44]. The coupling effect of the light in the adjacent waveguides forms a large number of subwavelength-scaled spots with similar optical-field distributions. By controlling the input laser power, nanoparticles with sizes from 100 to 500 nm were sorted based on the differentiated energy depths of each potential well. When sorting 200 nm nanoparticles, the trapping efficiency reached T>95% with P≥1.2 mW and v≤16 μm/s. High sorting efficiency and speed facilitate the simultaneous capture of large numbers of particles and the study of their specificity. The same team used coupled pore arrays to control the torsional moment exerted by adjacent pores on rod-shaped particles in 2019, as shown in Figure 7d [134]. Spherical bacteria become trapped in a single potential well because their dimensions are similar to those of a single potential well. However, rod-shaped bacteria are longer than single potential wells and subjected to the torque applied by the adjacent potential well. As a result, spherical *S. aureus* are separated from rod-shaped *E. coli* with similar volumes. When the velocity is increased to 14 μm/s, and the output power is 2 mW, the trapping efficiency of *E. coli* is more than 90%. This method overcomes the technical difficulties of separating bacteria of different shapes with similar volumes, and offers a solution for the shape-selective sorting of submicron particles.

### 3.4. Antigen/Antibody Interactions

Antibodies are among the key components of the humoral adaptive immune response, which can be used to neutralize and destroy pathogens. Upon initial exposure to a foreign antigen, the immune system generates a variety of polyclonal B-cell responses. Next, a series of initial antibodies are produced to recognize multiple overlapping and non-overlapping antigenic epitopes [135,136]. Indeed, due to the high specificity and the modular and adaptive structure of antibodies, as well as the availability of standardized manufacturing platforms, antibodies have become well established means of specifically recognizing antigens. Conversely, antigens can be means of specifically recognizing antibodies. When microspheres manipulated by OTs bind to the antigen/antibody due to specific antigen-antibody interactions, OTs can indirectly capture antibodies/antigens.

**Figure 8:**
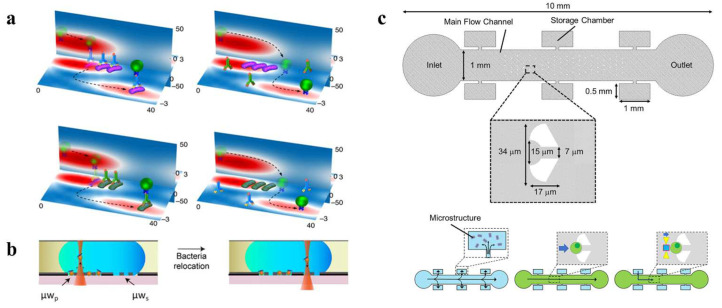
(**a**) Screening for bacterial antibodies with optofluidic tweezers with specific antibodies, reproduced with permission from [35]. Copyright @ 2018 Springer Nature. (**b**) Trapping and relocation of MBs with single bacteria from ${\mu W}_{p}$ to ${\mu W}_{s}$ with OTs, reproduced with permission from [138]. Copyright @ 2020 MDPI. (**c**) Principle of a cellular assembly platform in a microfluidic device, reproduced with permission from [139]. Copyright @ 2023 John Wiley & Sons, Inc.

In recent years, a number of researchers have greatly improved the specificity of optical capture and recognition using optofluidic OTs and antigen–antibody interactions. Jiang et al. developed a lab-on-a-chip device with rapid antibody-determination ability, using nanobeads as solid carriers [137]. The device includes an optical conveyor consisting of gold nanobeads in a microfluidic channel. When exposed to a uniform electric field, the polarization direction of the nanobeads labeled with the antibodies aligns with the field. The hot spots in the conveyor then act as OTs, capturing and directing the labeled bead in a specific direction. Only the specific antigens that bind to the antibodies tested can be dragged by the larger nanobeads and transported with them. Thus, the antibodies can be determined experimentally by identifying the fluorescently labeled nanobeads at the exit of the main channel. With the ability to detect in parallel, this design provides an attractive solution for the rapid, high-throughput determination of antibodies in microfluidic channels. Shi et al. revealed a multi-particle hopping mechanism between optical potential wells based on Kramer’s single-particle hopping theory, as shown in Figure 8a [35]. By adjusting the optical force and the fluid drag force in the microchannel, particle hopping between optical spots can be precisely controlled. Individual bacterial cells were trapped in the optical potential well. When conjugated with their specific antibodies, they bound to the microparticles that passed by and hopped away from the potential well. For antibodies with high specificity, only the target bacteria hopped away with the microparticle, whereas the non-target bacteria remained trapped. By calculating the proportion of hopping bacteria, the efficiency of the bacterial binding to antibodies can be measured at the single cell level. This provides a new platform for studying the interaction between biological particles. In 2020, Kumar et al. integrated digital microfluidics (DMF) with OT to selectively capture, reposition, and further proliferate individual bacterial cells while providing the continuous imaging of cells to assess dynamic cell behavior (Figure 8b) [138]. Magnetic beads coated with Salmonella typhi targeting antibodies are immobilized in the microwell array of the DMF platform and used to capture single cells in the fluorescent Salmonella typhi population, which can be followed by OTs to select a bead with the target bacteria and transfer it to a different microwell. The OT-integrated DMF platform successfully achieves the selective capture, retrieval, and repopulation of bacteria of interest through single-cell-level localization and proliferation, and allows further downstream analysis.

In addition, optofluidic tweezers incorporating antigen–antibody interactions can perform some specific tasks due to the specificity and efficiency of antigen–antibody interactions. In 2023, Mori et al. developed a platform for in situ cell assembly in a microfluidic device using optically driven micro-tools (Figure 8c) [139]. Antibodies immobilized on the micro-tool bind to antigens on the cell membrane and are captured by the micro-tool. Experiments demonstrate that multiple cells are assembled into a cell cluster by a repetitive cell-capture process. The geometry and surface function of the micro-tool can be modified to enhance its adaptability for different applications according to the cell-assembly requirements. This platform can be used in regenerative medicine and drug screening with the targeted generation of cell clusters.

### 3.5. Raman-Assisted Optical Tweezers

Raman spectroscopy is a light-scattering technique that utilizes a laser-light source to scatter high-intensity incident light through molecules. However, most of the scattered light has the same wavelength (color) as the incident laser, referred to as Rayleigh scattering, and cannot provide useful information. Only about $1∕10^{9}$ of the scattered light’s wavelength is different from the incident light; this is called Raman scattering. The wavelength change is determined by the chemical structure of the test sample, which is the so-called scattering material. A Raman spectrum typically consists of several Raman peaks, with each peak representing the corresponding wavelength position and the intensity of the Raman scattering light. Each peak corresponds to a specific molecular bond vibration, including not only a single chemical bond but also the vibrations of groups composed of several chemical bonds. 

**Figure 9:**
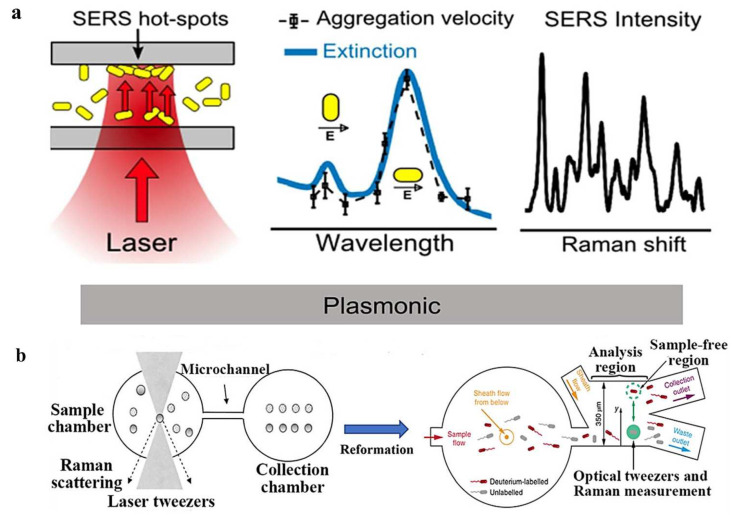
(**a**) Raman-assisted OTs are used to induce aggregation and detect the aggregation of metal nanoparticles (AuNR), reproduced with permission from [37]. Copyright @ 2019 American Chemical Society. (**b**) Screening of living cells with corresponding Raman spectra using Raman-assisted OTs, reproduced with permission from [142]. Copyright @ 2019 Springer Nature.

Raman spectroscopy is an excellent tool for performing the principal component analysis for materials, and it has been adequately integrated with OTs. This integration remarkably raises the level of understanding of laser-spectroscopy measurements for living cells or particle aggregates. The high biocompatibility of OTs can be combined with the Raman technique to provide structural and conformational information about chemical bonds, and to enhance the analytical and sorting capabilities of OTs. Some scholars prefer to refer to this process as “Raman tweezers”. In 2018, Pilát et al. utilized OTs to study the phenotypes of individual *E. coli* cells in isolation using a microfluidic chip with microchambers [36]. The OTs served to transfer individual cells from the main channel to the microchamber, as well as to immobilize the cells during the characterization of the phenotype performed. The group investigated the contact of cells with cefotaxime in combination with Raman spectroscopy and demonstrated the ability to culture individual cells in real time, as well as providing direct estimates of the therapeutic effects of antibiotic drugs. In 2019, Bernatová et al. demonstrated an experimental approach to obtain surface-enhanced Raman spectroscopy (SERS)-activity hotspots directly within the microfluidic chip, as shown in Figure 9a [37]. The use of optical forces to create aggregates of metallic nanoparticles allows the Raman signal to exceed the common detection limit, and for proteins down to the concentration of pM to be reliably detected ultra-sensitively in a short time. Dai et al. developed an in situ optical-tweezer-coupled SERS method that facilitates the dynamic detection of transient species of a-synuclein at physiological concentrations of 1 mM in 2021 [38]. This experimental approach directly characterizes the structures of proteins without disturbing their original state, and is expected to address the structural details of dynamic, heterogeneous, and complex biological systems in the future.

Automated Raman-tweezer-based cell-sorting platforms have emerged that include a multi-channel microfluidic chip for automated delivery, manipulation, analysis, and sorting of single cells in continuously flowing samples [140]. Casabella et al. built the first fully automated Raman-assisted sorting system in 2016, in which Raman and capture lasers, panning tables, and syringe pumps were all managed by one type of software [141]. The batch characterization of 143 cells for Raman spectroscopy in 50 min was demonstrated in their experiments. In 2019, Lee et al. developed a customizable, fully automated optofluidic platform for Raman spectroscopy, which isolates cells through single-cell Raman spectroscopy and decision criteria without human intervention, as shown in Figure 9b [142]. This sorting method can achieve the high-precision classification of model bacteria (98.3% ± 1.7%) and relatively high throughput (3.3–8.3 cells/min) at the single-cell level. The investigators applied this sorting method to the macrogenomic characterization of bacteria involved in mucin degradation in mouse colons, highlighting the potential of Raman-activated cell sorting in the identification of key players in the targeting process.

### 3.6. Chiral Optical Forces

Optical lateral forces induced by a linearly polarized laser beam are predicted to deflect bipolar particles of opposite chirality in opposite lateral directions. The “chirality-dependent” forces, called optical lateral forces, may offer new possibilities for the passive all-optical selective sorting of chiral particles. Optical lateral forces are perpendicular to the direction of light propagation and have a unique advantage in controlling chiral particles. However, in experiments, it is often overshadowed by stronger gradient forces, making it difficult to control chiral particles alone [23,143].

**Figure 10:**
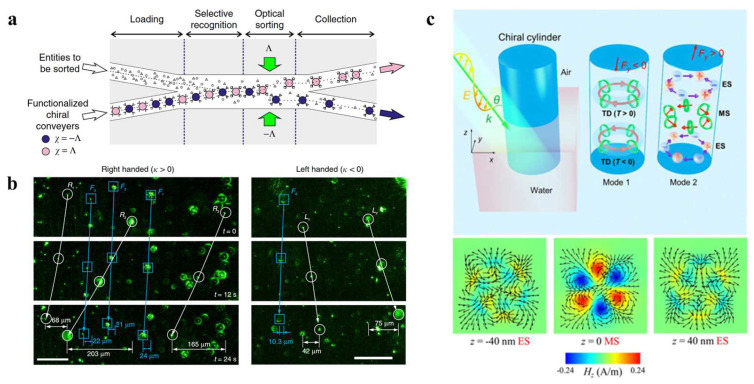
(**a**) Using different scattering forces of different chiral particles to separate different chiral particles in microchannels, reproduced with permission from [144]. Copyright @ 2014 Springer Nature. (**b**) Chiral particle separation is achieved by transverse momentum migration, reproduced with permission from [43]. Copyright @ 2020 Springer Nature. (**c**) Composite multipole mode greatly enhances optical lateral forces, reproduced with permission from [145]. Copyright @ 2020 American Physical Society.

In recent years, the means of controlling optical forces in experiments have advanced considerably. Optical transverse forces can be clearly observed when the control gradient force approaches 0. The asymmetric manipulation and sorting of chiral particles can be achieved using chirality-related optical transverse forces in the presence of controlled other forces. Tkachenko et al. experimentally demonstrated the passive optical separation of mirror-image chiral microparticles with only opposite chiral differences in a fluidic environment using chiral light fields, as shown in Figure 10a [144]. Importantly, the proposed optofluidic strategy can also be used without chiral-shaped microparticles and actually depends on the chirality of the surrounding medium. In 2020, Shi et al. revealed an asymmetric transverse momentum-transfer mechanism through the interaction of light and micrometer-sized chiral particles placed at the air–water interface, as shown in Figure 10b [43]. Contrary to conventional theory, the direction of the transverse force depends not only on the chiral state of the particle, but also on the polarization of the light, the angle of incidence, and the chiral state of the particle. The gradient force is effectively weakened by using band-line polarized light to eliminate its interference with the transverse force, enabling the optical transverse force to move particles in opposite chiral states in opposite directions, thereby separating them.

In addition to minimizing the influence of other optical forces, the direct enhancement of optical lateral forces can also be used. Zhu et al. demonstrated that composite multipole modes can be used to significantly increase chiral lateral forces by hundreds of times and raise them to the same order of magnitude as the scattering force through coupling and conversion between different modes (Figure 10c) [145]. This has important implications for the enhancement of chiral optical force and the separation of chiral particles. 

### 3.7. Other Optofluidic Tweezers 

**Figure 11:**
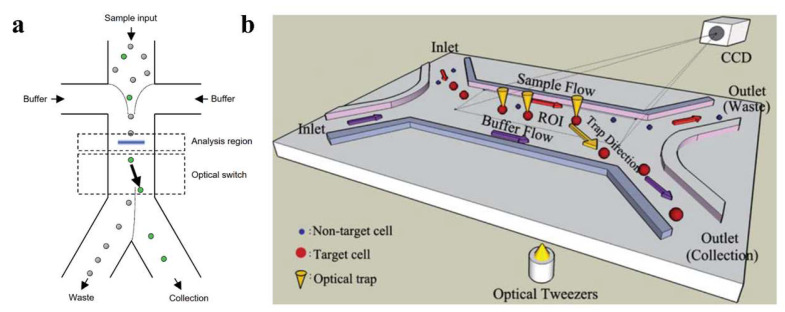
(**a**) Schematic diagram of a device for sorting GFP-expressing HeLa cells, green circles indicate target cells, while gray circles indicate other cells, reproduced with permission from [19]. Copyright @ 2005 Springer Nature. (**b**) A type of optical switch based on single-mode fiber (SMF) in microfluidic channel, reproduced with permission from [147]. Copyright @ 2011 The Royal Society of Chemistry.

An optical switch can obtain information from different cells through various measurement methods and use OTs to selectively transfer target bioparticles to specific channels for cell sorting. In 2005, Wang et al. designed an optical switch capable of selectively irradiating with a laser beam by detecting the fluorescent signals of different cells. Subsequently, optical scattering force was used to push the specified cells to a specific exit channel (Figure 11a) [19]. This method uses a laser energy of more than 10 W and permits cell screening at high flow rates, of 25 mm/s. However, high-powered, high-energy-density lasers may be damaging to biological samples and are therefore restricted in practical applications. Lin et al. proposed a novel microfluidic system that combines computer-controlled digital image processing (DIP) technology with OTs for the automatic identification, counting, and sorting of cells/micro-particles in a continuous flow environment [146]. In the proposed system, cells/micro-particles are electrokinetically focused into a narrow sample stream, identified, and tracked in real time using a proprietary DIP system as they are driven through the region of interest. A focused infrared laser beam, driven by a synchronized control signal generated by the DIP system, transfers target cells from the main sample stream to an adjacent sheath stream. Subsequently, the target cells are transferred by the stream to the collection channel, where they are automatically counted. The microchip was demonstrated to be capable of continuously sorting and counting microparticles with diameters of 5 and 10 μm. This system provides a simple, low-cost, high-performance solution for cell manipulation in microfluidic devices. In 2011, Wang et al. developed a versatile single-cell-manipulation tool based on dynamic fluid and dynamic light modes, as shown in Figure 11b [147]. This technology employs both single and multiple laser traps to transport cells with high accuracy and recognize multiple cell features, such as their size and fluorescent labeling, to process small cell populations. Furthermore, OTs are used to move target cells to desired destinations non-invasively and precisely. The image-processing method incorporated into this tool provides a high level of precision for the sorting of small cell populations. In experiments involving the sorting of yeast cells and human embryonic stem cells, recovery and purity rates of over 90% were achieved. Xu et al. introduced an optical-tweezer-assisted microfluidic cell screening and single-cell isolation (OPSI) device for capturing, manipulating, encapsulating, and outputting individual cells in the size range of 1 to 40 μm [148]. The OPSI accurately indexes cells for classification based on real-time imaging with high-resolution fluorescence, bright-field microscopy, or Raman maps. Tests on bacteria, yeast, and human cells (ranging from 1 to 40 μm in diameter) showed that the target cells were sorted with a purity >99.7% and at a rate of 10−20 cells per minute. The versatility, convenience, flexibility, and low cost of the OPSI make it a promising tool for image-based research.

Optical chromatography is a method in which weakly diverging Gaussian light produces attenuating optical forces in a fluid environment. Optical forces, along with fluidic forces in the opposite direction, capture particles of different sizes or with different refractive indices at different locations. The weakly diverging Gaussian light, with a gradient in the direction of propagation, results in the exertion of linear force on the particles, depending on their sizes. Therefore, particles of different sizes are captured at different positions within the beam by achieving a balance between fluidic and optical forces. The forces on particles can be computed using a damping-system model that provides mechanical insights into particle motion. Nan et al. produced versatile and tunable optofluidic potential traps by synchronizing phase-gradient forces and fluidic drag forces, enabling the controlled trapping, sorting, and transport of metallic NPs below 100 nm in diameter [149]. The potential energy distribution of metallic NPs can be precisely designed by tuning the phase gradient, laser power, and flow rate of the quasi-one-dimensional optofluidic trap. 

There are various techniques for micro and nano manipulation with optofluidic tweezers. Shi et al. studied a damping diagram through the synchronization of optical and fluidic forces for particle manipulation and separation [150]. The “loosely overdamped system,” which means an overdamped system with low optical-trap stiffness, was found to separate nanoparticles of different sizes with nanoscale precision. Gold nanoparticles with radii from 30 to 50 nm were separated with an accuracy of 5 nm, holding promise for the precise sorting of small particles, such as bacteria and viruses. Additionally, the vibration amplitudes of the particles in the loosely overdamped system were two orders of magnitude higher than those captured by conventional OTs. Wu et al. used impinging streams to create a stagnation point in a flowing system, in which nanoparticles with different sizes can be separated by a laser beam with high throughput [31]. Zheng et al. integrated near-infrared OTs with upconversion luminescence encoding to demonstrate a specialized microfluidic-chip-assisted platform [151]. They achieved the automated and simultaneous quantification of miRNA-205 and miRNA-21 sequences with a detection limit at the pM level. Moreover, the technique was successfully used to analyze complex biological samples, such as cell lysates and human-tissue lysates, suggesting potential in disease diagnosis. In 2020, Yao et al. used fluidic drag forces to stretch cells in a microfluidic channel transverse to optical tweezers [33]. This design allows cell stretching and continuous cell delivery, as well as the characterization of the mechanical properties of cell surfaces to distinguish certain lesions in cells. It has considerable potential for medical monitoring and biomechanical studies.

## 4. Summary and Outlook

The combination of OTs with microfluidics has been applied to develop optofluidic tweezers, which have achieved great success in micro/nano manipulation. Optofluidic tweezers reshape static environments into dynamic and accurately controlled environments. Microfluidics-based high throughput has greatly improved the number and efficiency of manipulating particles. These OTs can quickly capture any particle in the flow and measure its characteristics with high precision. 

While optofluidic tweezers have displayed remarkable achievements, there is still significant potential for improvement in many respects. For example, most particle-screening techniques handle small-volume samples, usually in the order of nanoliters or below. Further increases in throughput usually require extremely high laser power, such as several watts or higher. When the throughput is high (no less than 1000 particles per minute), for accurate sorting, the size limit of the target particle is usually about 200 nm, and further decreases in the particle size greatly reduce the screening speed and accuracy [23]. Technologies capable of the high-speed and high-throughput screening of nanoparticles are urgently needed for many biomedical applications, such as the efficient separation of viruses and exosomes in bodily fluids and the detection of diseased cells in the blood. To accomplish this task, the optical and fluidic fields must be optimized simultaneously to obtain greater optical forces to perform particle manipulation in a more rapidly flowing system.

At present, chiral optical forces are generally small, and most of the research on these forces is still in the theoretical stage. In addition, the mechanism related to the coupling of fluidic and optical force is not fully understood. In most studies, fluid is only used as a transmission medium, and the degree of freedom of the system caused by fluidic force has not been fully developed. New screening or measurement techniques, such as the interaction between antigens and antibodies to improve the specificity, are highly necessary. With the development of nanofabrication technology and micro-nano optics, light-driven microrobots have received widespread attention for their potential in various biomedical applications [152]. In the future, multiple-degree-of-freedom light-driven microrobots could be designed for practical applications, such as drug delivery, mixing, and cancer targeting.

Finally, there have been many studies in recent years using software for image recognition, environmental control, and OT manipulation, while research on the combination of optofluidic tweezers and deep learning is still in its early stage. Further developments in this area may greatly reduce the cost and boost the manipulation speed of optofluidic tweezers. 

In the foreseeable future, optofluidic tweezers will be more widely used in biomedical and physical scientific fields, playing a pivotal role in diverse schemes, such as improving efficiency and utility in cell therapy and clinical diagnoses. However, it is crucial to acknowledge that the resolution of the aforementioned challenges, for which optofluidic tweezers hold great potential, hinges upon breakthroughs in the corresponding technologies. Only through these advancements can we expect significant progress and developments in the field. This will take significant time. Although there have been increasing examples of the combination of optofluidic tweezers with other technologies in recent years, artificial intelligence and deep learning may enable further developments of this method. The combination of these technologies with optofluidic tweezers is still in its infancy, and this may become one of the bottlenecks in the development of optofluidic tweezers. This is an urgent issue that needs to be addressed. Moreover, with the development of other technologies, especially in the biological and medical fields, some techniques have emerged that can partially replace optofluidic tweezers, such as flow cytometry. Due to its ubiquitous use and low cost, flow cytometry is playing an increasing role in the biological and medical fields. Researchers are required to improve the uniqueness and irreplaceability of optofluidic tweezers, which is a serious challenge.

## Data Availability

Not applicable.
